# Tube length of chironomid larvae as an indicator for dissolved oxygen in water bodies

**DOI:** 10.1038/s41598-022-23953-9

**Published:** 2022-11-19

**Authors:** Rahul Podder, Susanta Nath, Biplob Kumar Modak, Lennart Weltje, Biswanath Malakar

**Affiliations:** 1 Department of Zoology, Sidho-Kanho-Birsha University, Purulia, West Bengal 723 104 India; 2 Department of Zoology, Government General Degree College Singur, (University of Burdwan), Hooghly, West Bengal 712 409 India; 3 BASF SE, Agricultural Solutions-Ecotoxicology, Speyerer Strasse 2, 67117 Limburgerhof, Germany; 4 Department of Anthropology, Government General Degree College Singur, (University of Burdwan), Hooghly, West Bengal 712 409 India

**Keywords:** Ecology, Zoology

## Abstract

Tube-building larvae of non-biting midges, or chironomids, are considered bioindicators of water pollution. The larvae use benthic particles to make their tubes and create a respiratory current with the movement of their bodies inside the tubes. The tube length of the chironomid larvae varies depending on several physicochemical properties of the aquatic medium. Here we study the role of physicochemical parameters on the tube length from different field sites and in the laboratory. It appears that among different physicochemical factors, dissolved oxygen (DO) has a major role in determining the tube length of the larvae. A quantitative relationship between oxygen concentration and the tube length of larvae is presented here. Our study reveals a longer tube in aquatic media with oxygen deficiency and a shorter tube in those with higher oxygen. This result may help to assess the quality of water bodies and, in particular the status of DO.

## Introduction

Chironomids (Diptera: Chironomidae) play a major role in the aquatic food chain by maintaining a link between producer and primary carnivores. They are also considered as important biological tools for environment assessment. Chironomids are primary consumers and a source of food for the higher trophic levels^1^. Tube building is an important characteristic feature of chironomid larvae and the tubes protect the larvae from the effects of heavy metals and other pollutants to some extent^2,3^. Larvae make their tubes with salivary silk and soft benthic particles^4^. The composition of the tube of the larvae is similar to the benthic particles of the surrounding medium. The quality of building materials is more important than the size of particles, in upstream of lotic water^5^. Tubes help to collect food particles, assist larvae in producing respiratory currents, act as a refuge from predators and inhibit chemical toxicants^6–8^. Halpern et. al. reported tubes of these larvae protect them from predation by damselflies^9^.Tube building properties also can modify the quality of the substrate used for tube building^10^. As a bio-indicator species, larvae are used to understand the quality of the aquatic body. *Chironomus plumosus* and *Polypedilum* sp. have a great influence on de-nitrification and nitrogen turnover in lake deposits^11^. Different physicochemical factors such as water temperature, alkalinity, and benthic soil organic carbon have been found to influence the seasonal variation of body protein and the growth of chironomid larvae^12,13^. At the same time, some environmental parameters also influenced the tube-building ability of the chironomids. During the study, a strong relation between the tube length and DO was observed. As well as this feature will help the person who is involved in freshwater aquaculture to measure the DO by a standard scale without using any expensive instrument or titrimetric method. Keeping these features in mind, here an attempt has been made to find out the relationship between tube length and dissolved oxygen (DO).The work seems to be unique and novel for its own kind.

## Materials and methods

The Kanchrapara wastewater canal (KWC) (22°93 N, 88°47 E) and Kanchrapara fish culturing pond (KFP) (22°94 N, 88°45 E), Kanchrapara, North 24 Parganas, West Bengal, India, are chosen for this experiment in natural conditions to study the relationship between the tube length of chironomid larvae and different physicochemical parameters of the aquatic bodies. The distance between two water bodies is approximately five kilometres, and these water bodies may be an ideal model for the urban aquatic ecosystem due to their different quality of water. KWC is enriched with a high organic and nutritive content. Because of the fish culture in KFP, nutrient quality is also maintained in a controlled manner. In KFP, *Wolffia* sp. and *Lemna minor*, are observed mainly during the monsoon as floating vegetation. Characteristics of the aquatic bodies are presented in Table 1.

**Table 1:** Dimensions of sampling sites.

SITE	Length (m)	Width (m)	Perimeter (m)	Area (m^2^)	Shape	Altitude (m)
KWC	447	3.5	805 (2 side)	1564	Linear	12
KFP	332	220	993	63,560	Rectangular	8

*KWC* Kanchrapara wastewater canal, *KFP* Fish culturing pond of Kanchrapara.

Physicochemical parameters like DO (dissolved oxygen), free CO_2_, water temperature, salinity, hardness, alkalinity, ammonia, nitrate, nitrite, iron, and benthic soil organic carbon are measured from the collected water and benthic soil samples following the APHA methods^14^ between 2015 and 2019. To measure the tube length of the larvae of *Chironomus striatipennis* Kieffer (1910) tubes were kept on blotting paper and measured by a standard measuring scale during the abundance study of these two sites. *C. striatipennis* is considered a model chironomid due to its high abundance in both the study sites.

Based on the observations under natural conditions, laboratory experiments were conducted to observe the relationship between DO and tube length of *Chironomus* larvae. For the laboratory study, larvae of *Chironomus striatipennis*Kieffer (1910) are obtained from laboratory culture. Approximately 300 newly hatched larvae are placed into three experimental trays (TARSONS) (10ʺ × 8ʺ × 6ʺ) containing finely washed and autoclaved silt (@ 0.02 g soil per larva) at the bottom (Supplementary information 1). The larvae are reared up to the 2^nd^ instar stage with filtered pond water (2 inches high in the tray) and DO is maintained with an aquarium air pump. The larvae are acclimatised to the artificial conditions and have reached the second instar stage in their tube. At this point, water from the experimental trays is replaced with treated pond water. The pond water is collected from the study area during sample collection and DO is measured. Treatment (25%, 50%, 75% grade of dilution) is done by mixing distilled water (boiled and cooled) to confirm the minimization of oxygen concentration, as boiling removes dissolved oxygen from water. DO is measured by the Winkler method^15^. Three differently treated waters are poured into three separate trays carefully. Artificial aeration is turned off and trays are covered with a cotton cloth to minimise the oxygenation of water from the air.

The control is maintained in another experimental tray following the same protocol with untreated pond water. DO is maintained by the aquarium air pump.

The level of DO and the tube length of the larvae are monitored at every 24-h interval for 7 consecutive days in both control and treated trays. The experiment was replicated twelve times.

### Statistical analysis

To investigate the effect of aquatic physicochemical parameters on tube length, stepwise linear regression analysis is performed, considering tube length as a dependent variable while all aquatic physicochemical parameters are independent variables. In the step-wise method, the last model is considered with its highest R^2^ value along with standardised coefficients (β) of different parameters (Supplementary information 2). R^2 ^is a statistical measure that represents the proportion of the variance for a dependent variable i.e. explained by the independent variable (s) in a regression model. A simple linear regression along with ANOVA was also performed to justify the relationship between DO and tube length of the larvae in both natural and laboratory conditions. A graphical model is also constructed for a more straightforward interpretation of the result. Statistical analyses are done using Microsoft Excel (2016) and SPSS software version 26 (SPSS Inc., Chicago, IL, USA).

## Results

The stepwise linear regression revealed a significant negative relationship between tube length and DO (Model IV: β = − 2.779, Model VI: β = − 2.044; p < 0.001). A positive relation between tube length and free carbon dioxide (F CO_2_) also exists. Water temperature (W TEMP) and total hardness (TH), nitrate and iron of the water also shows the relationship with the tube length. But the priority is given to DO because of its high negative relation with tube length in both models (Table 2).

**Table 2:** Stepwise linear regression analysis between tube length (dependent variable) and aquatic physicochemical parameters (independent variables).

Model	Standardized coefficients (β)	Standard error (SE)	p-value
**IV**			
Constant	27.291	0.667	< 0.001
DO	− 2.779	0.087	< 0.001
W TEMP	− 0.215	0.014	< 0.001
F CO_2_	0.028	0.006	< 0.001
Nitrate	− 1.259	0.373	< 0.001
R^2^	0.996		
**VI**			
(Constant)	8.029	0.841	< 0.001
DO	− 2.044	0.195	< 0.001
F CO_2_	0.41	0.01	< 0.001
TH	0.051	0.011321	< 0.001
Iron	− 1.296	0.511	< 0.001
R^2^	0.986		

During the study period, the DO values of these two aquatic bodies were recorded in their natural state (Tables 3 and 4).

**Table 3:** Seasonal variations of DO and tube length of chironomid larvae of Kanchrapara wastewater canal.

	Pre monsoon	Monsoon	Post monsoon	Winter
**2015–2016**				
DO	3.66–4.28	4.41–5.39	6.21–6.41	6.3–7.6
	3.94 ± 0.25	4.91 ± 0.45	6.02 ± 0.40	6.76 ± 0.59
Tube length (mm)	8.1–8.7	7.1–7.85	7.9–8.9	7.9–8.2
	8.43 ± 0.30	7.58 ± 0.41	8.53 ± 0.55	8.06 ± 0.15
**2016–2017**				
DO	3.6–4.41	3.9–5.0	5.03–5.4	5.63–6.74
	4.07 ± 0.34	4.5 ± 0.45	5.24 ± 0.15	6.1 ± 0.42
Tube length (mm)	9.5–10.1	8.3–9.8	7.8–8.1	7.7–8.2
	9.76 ± 0.30	9.1 ± 0.75	8 ± 0.17	7.9 ± 0.26
**2017–2018**				
DO	4.09–4.67	3.87–5.08	4.66–5.4	6.39–7.39
	4.34 ± 0.24	4.58 ± 0.51	5.03 ± 0.30	6.80–0.42
Tube length (mm)	9.2–8.8	10.2–8.6	10.3–8.3	7.8–7.1
	9.03 ± 0.20	9.43 ± 0.80	9.03 ± 1.10	7.5 ± 0.36
**2018–2019**				
DO	3.67–4.2	4.39–6.3	4.6–6.4	6.97–7.6
	3.85 ± 0.24	5.03 ± 0.89	5.42 ± 0.74	7.22 ± 0.37
Tube length (mm)	9.7–10.2	7.6–9.3	7.9–9.3	7.3–7.4
	10 ± 0.26	8.7 ± 0.95	8.43 ± 0.75	7.36 ± 0.05

Data is presented by range, mean and standard deviation.

**Table 4:** Seasonal variation in DO and tube length of chironomid larvae from fish culturing ponds of Kanchrapara (data presented as arange, mean and standard deviation).

	Pre monsoon	Monsoon	Post monsoon	Winter
**2015–2016**				
DO	5.15–6.65	7.67–8.29	5.93–7.96	7.3–8.4
	5.86 ± 0.61	7.95 ± 0.25	6.86 ± 0.83	7.89 ± 0.45
Tube length (mm)	7.2–8.5	7.1–7.5	7.8–8.1	7.2–7.9
	7.93 ± 0.66	7.33 ± 0.20	7.96 ± 0.15	7.63 ± 0.37
**2016–2017**				
DO	5.32–5.83	4.8–6.4	6.41–7.8	6.13–8.43
	5.53 ± 0.21	5.66 ± 0.65	6.88 ± 0.65	7.6 ± 1.04
Tube length (mm)	7.8–8	7.2–9.4	7.1–8.6	7.3–9.1
	7.9 ± 0.10	8.13 ± 1.13	7.6 ± 0.86	8.46 ± 1.01
**2017–2018**				
DO	4.12–6.1	5.2–5.97	5.16–8.9	6.19–8.67
	4.93 ± 0.84	5.68 ± 0.34	7.22 ± 1.55	7.92 ± 1.22
Tube length (mm)	6.9–9.2	6.8–7.4	6.8–8.1	7.0–7.6
	8.33 ± 1.25	7.13 ± 0.30	7.26 ± 0.72	7.33 ± 0.30
**2018–2019**				
DO	5.2–6.2	4.89–6.1	6.78–7.1	7.1–8.67
	5.57 ± 0.44	5.63 ± 0.53	6.92 ± 0.13	7.95 ± 0.64
Tube length (mm)	7.3–7.8	8.1–9.1	7.6–8.0	7.2–7.7
	7.6 ± 0.26	8.66 ± 0.51	7.8 ± 0.2	7.43 ± 0.25

Results are also recorded from the experimental setup in the laboratory condition along with control. After running the experiment for seven days, the amount of DO and length of the tubes of every experimental setup are considered (Table 5).

**Table 5:** Dissolved oxygen and tube length of chironomid larvae of different grades of diluted water in laboratory conditions along with control (data presented as range, mean and standard deviation).

Dilution	DO (mg l^−1^)	Tube length (mm)
Control	6.1–7.8	7.0–8.2
	7.03 ± 0.42	7.61 ± 0.31
A	4.5–5.9	7.9–8.9
	5.20 ± 0.43	8.48 ± 0.33
B	3.1–4.1	8.7–9.7
	3.55 ± 0.31	9.3 ± 0.30
C	1.5–2.8	8.9–10.4
	2.00 ± 0.38	9.91 ± 0.40

*DO* Dissolved oxygen, *A* 25% dilution, *B* 50% dilution, *C* 75% dilution.

**Figure 1:**
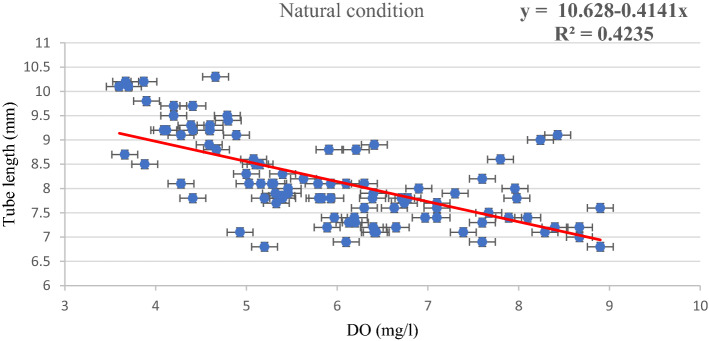
Linear regression of tube length against dissolved oxygen (DO) under natural conditions.

**Figure 2:**
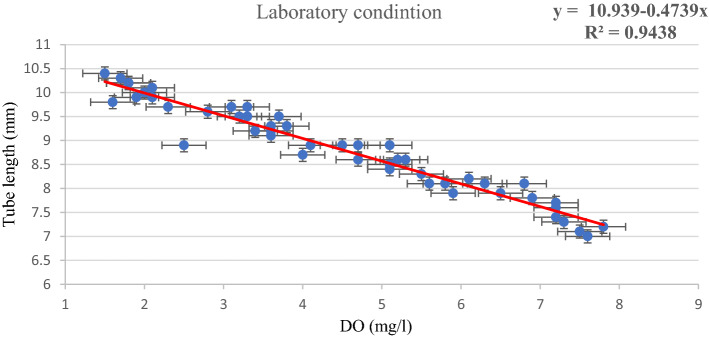
Linear regression of the tube length against dissolved oxygen (DO) under laboratory conditions.

A simple linear regression model is constructed between the DO (independent variable) and tube length (dependent variables) of the chironomid larvae in natural conditions as well as laboratory conditions (Figs. 1 and 2).

Under both natural and laboratory conditions (Figs. 1 and 2), a strong linear relationship is observed between the DO and the tube length. R (R for natural conditions: 0.650; R for laboratory conditions: 0.971) is the correlation coefficient value which is significantly high in both conditions. The R^2^ values (R^2^ for the natural condition: 0.423; R^2^ for laboratory condition: 0.943) indicate that 42% of the variance in tube length in the case of natural condition and 94% of the variance in tube length in laboratory condition can be accounted for by the dissolve oxygen measure. In both natural and laboratory conditions, the standard error is observed with a nominal value. The linear relationship is also confirmed by the significant *F* value (natural condition *F* = 69.04, *p* < 0.05; laboratory condition *F* = 772.81, *p* < 0.05). The scatter plot and linear regression both show significant inverse relationships between DO and larval tube lengths.

**Figure 3:**
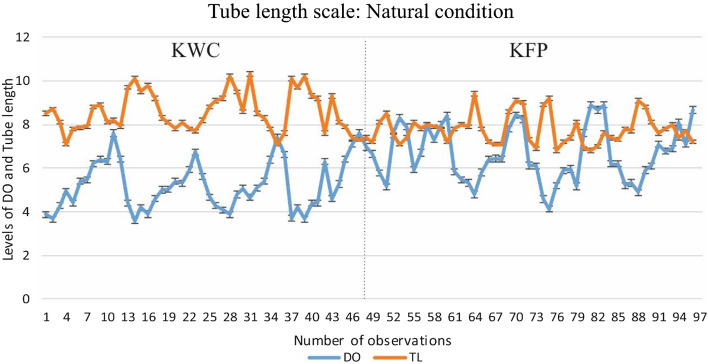
Graphical representation of the relationship between dissolved oxygen and tube length of *C. striatipennis* under natural conditions (*KWC* Kanchrapara wastewater canal, *KFP* fish culturing pond of Kanchrpara, *TL* Tube length).

**Figure 4:**
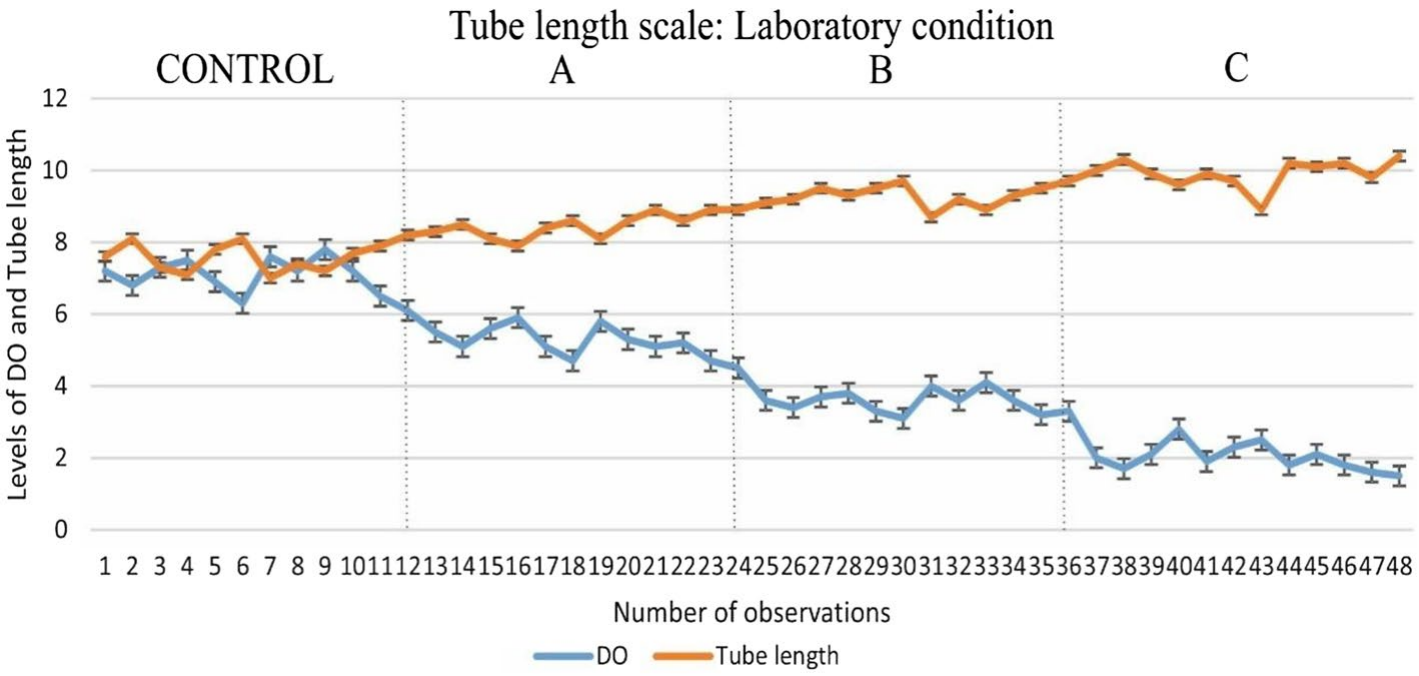
Graphical representation of the relationship between dissolved oxygen and tube length of *C. striatipennis* under laboratory conditions (A: 25% dilution, B: 50% dilution, C: 75% dilution).

The result showed that in all the cases, the tube length of *Chironomus striatipennis *increased with the decrease of DO. Since KWC is a wastewater canal, the fluctuation of oxygen in KWC is much higher than in KFP throughout the year. The effect of fluctuating DO on the tube length of larvae is higher in KWC than in KFP. Similar effects have also been observed in laboratory conditions. Graphical representations are made to compare the tube length and DO of water at the two study sites and also with the laboratory data (Figs. 3 and 4).

## Discussion

Chironomids have the ability to survive and reproduce in polluted environments, and thus they are included in many ecological studies where approaches may be taxonomic or functional^16^. The diversity of most macroinvertebrates is controlled by the oxygen level of water, but chironomids may survive in hypoxic conditions where the oxygen concentration may be less than 3 mg l^−1^^17^. The current study demonstrates that changing seasons, as well as anthropogenic activities, have a significant impact on the levels of DO in aquatic bodies. As observed from the result, DO highly influences the tube length of the chironomid larvae. Since KWC is a wastewater canal, the average oxygen level is lower (5.24 ± 1.14 mg l^−1^) than KFP (6.63 ± 1.28 mg l^−1^) which is a normal fish culturing pond. It has also been observed that the average tube length of the chironomid larvae of KWC (8.66 ± 0.88 mm) is higher than KFP (7.68 ± 0.62 mm), which indicates that a low concentration of DO promotes the building of longer tubes in natural conditions. Similar observations were also observed in laboratory conditions. When the oxygen level (7.03 ± 0.41 mg l^−1^) in the experiment was kept in the normal range, there was negligible variation in tube length (7.61 ± 0.31 mm). But when the concentration of oxygen is gradually reduced by dilution, the tube length starts to increase accordingly, which is explained graphically in Fig. 4. The regression model of both the experimental conditions also supports the hypothesis that the tube length has an inverse relationship with DO. The scatter plot and simple linear regression confirmed the inverse relationship between DO and tube length (Figs. 1 and 2).

Chironomid larvae are able to grow in the polluted water of a wastewater pond as dominant macroinvertebrates^18^. It is observed that those larvae living in the sand tubes are more susceptible to chemical pollutants than the larvae living in silt tubes^7^. Sand particles are bigger than silt and are not suitable for the survival of larvae^19^. *Chironomus riparius *larvae make their tubes from different external particles and their own proteins^20^. Midge larvae are the inhabitants of sediments, and at the same time, sediment is the depository of different inorganic, organic, and heavy metals. In such cases, the tube of chironomid larvae may act as a defensive structure, which protects them from the adverse effects of undesirable pollutants and may increase their tolerance against such chemicals^21–23^.

Larvae can thrive in benthic sediments with high decaying organic content and very low DO concentrations in water bodies^24^. In poor DO concentration, larvae can survive due to the presence of haemoglobin in their body tissue fluid, which plays an important physiological role in increasing respiratory efficiency, as was observed in *Chironomus plumosus*. Longer tube length may help larvae generate better respiratory currents so that they can cope with a low DO environment.

Tube length is crucial for living in water because primarily tubes protect them from outer environmental factors like predators, and pollution. It was observed during this study that when the DO of water is low, larvae make elongated tubes to reach the upper layer of water, where the DO level is comparatively high. To get their required amount of oxygen, the larvae increase the tube length towards the water surface and increase the DO in tube water by undulating the body and other structures, creating a current inside the tube^25,26^. On contrary, when the DO level of the surrounding water of chironomid is sufficient, they can manage their normal physiological activities with the available oxygen. They need not to elongate their tube length. That’s why their tube length is inversely related to the DO of their surrounding medium.

If tube length does not increase in size in hypoxic water, larvae will not be able to meet their oxygen demand. If the DO of water decreases, tube length will increase and vice versa. Behavioural and physiological adaptations of chironomids larvae make them successful to live in a hypoxic environment. Thus, in hypoxic conditions, larvae with longer tubes are able to gather more oxygen from the upper layer of water and get more space to create a current of water to increase the amount of O_2_ inside the tube by undulating the preanal papillae, anal gill, ventral gills. This would explain why the tube length of chironomids depends on the DO of water. Hence by measuring the tube length with a standard measuring scale, one may get an idea about the quality of water, especially DO, before doing any chemical analysis. The work seems to be unique and novel for its own kind.

## Conclusion

The role of physicochemical parameters of water on the tube length of chironomid larvae is considered in the present study. Among different parameters, dissolved oxygen has a major role in determining the tube length of larvae. Under both natural and laboratory conditions, a strong linear relationship is observed between the DO and the tube length. The value of R (correlation coefficient) is high both in natural as well as in laboratory conditions (0.650 and 0.971 respectively). The present study reveals a longer tube in aquatic media with the hypoxic condition and a shorter tube in those with normal DO in the surrounding medium. This result may help to assess the quality of water bodies and, in particular the status of DO. By measuring the tube length with a standard measuring scale, one may get an idea about the approximate level of DO in the aquatic bodies, without doing any chemical analysis or using the costly instrument (Supplementary Information).

## Supplementary Information

Supplementary Information 1.

Supplementary Information 2.

## Data Availability

All data used in this manuscript are original and collected from field and laboratory studies.
