# Supplementary material for: Tube length of chironomid larvae as an indicator for dissolved oxygen in water bodies
Source: Sci Rep. 2022 Nov 19;12:19971. doi: 10.1038/s41598-022-23953-9 (PMC9675818; doi:10.1038/s41598-022-23953-9)
Supplement: Supplementary file 1 — Supplementary Information 1. [file 41598_2022_23953_MOESM1_ESM.docx]

Photographs of *Chironomus striatipennis*


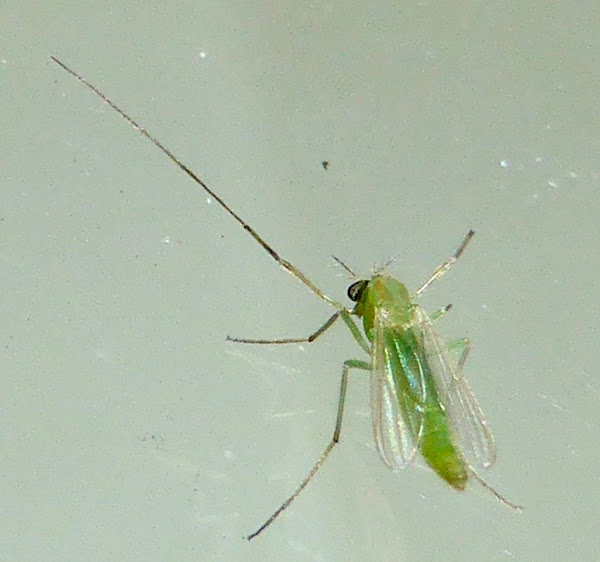


Photo S1: Enlarged view of adult female *Chironomus striatipennis*


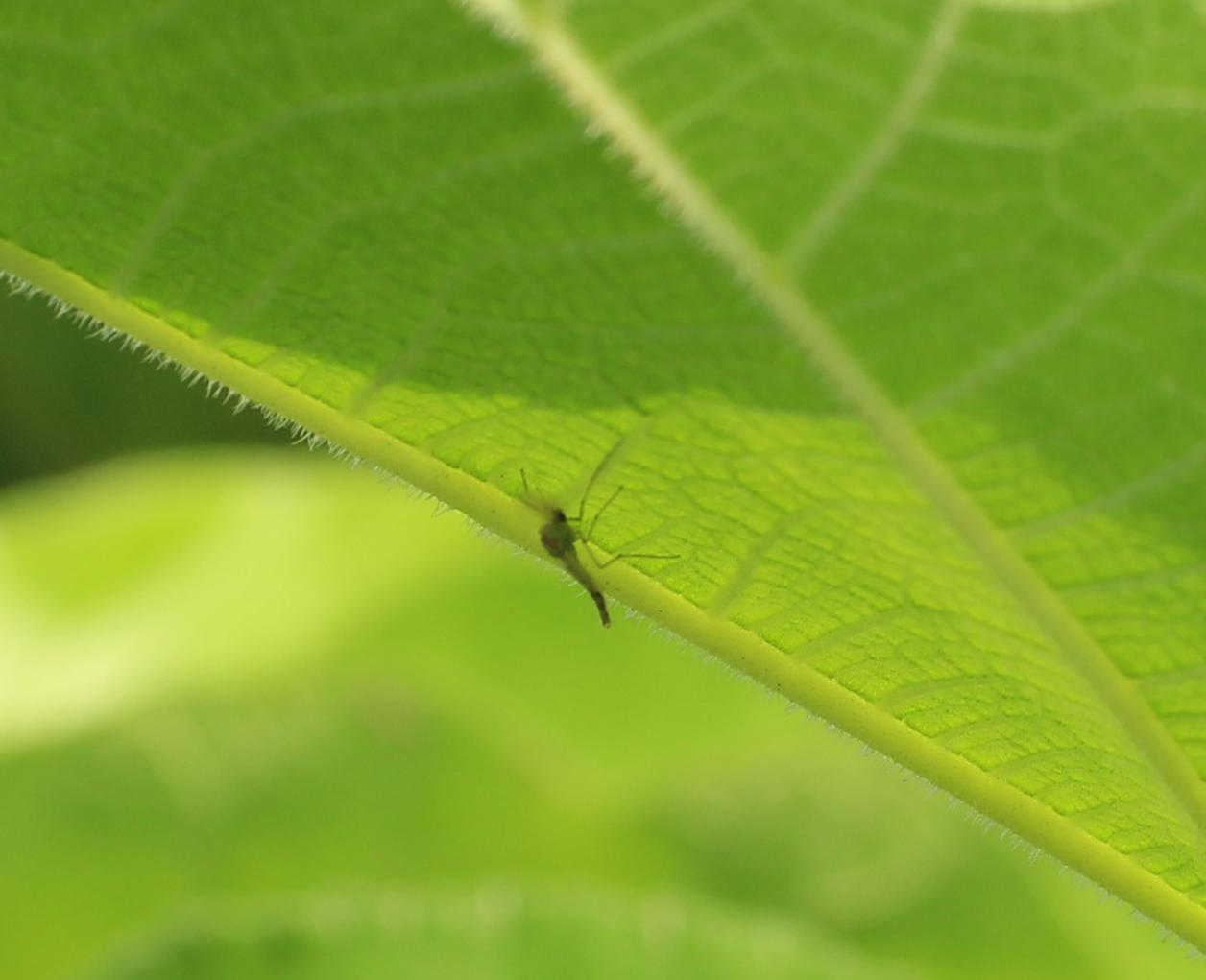


Photo S2: Adult male *Chironomus striatipennis* in their natural habitat


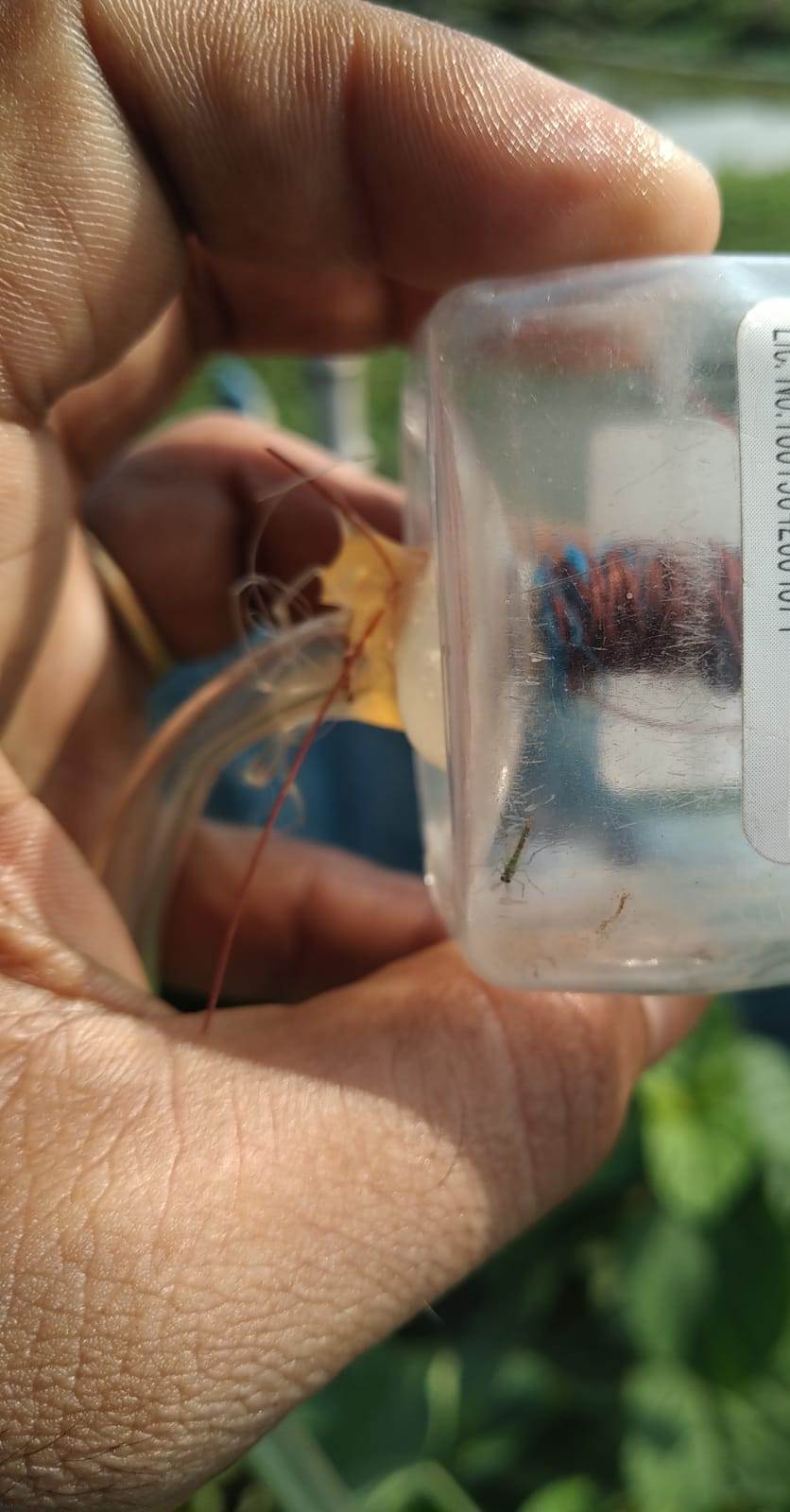


Photo S3. Collection of Adult *Chironomus striatipennis*


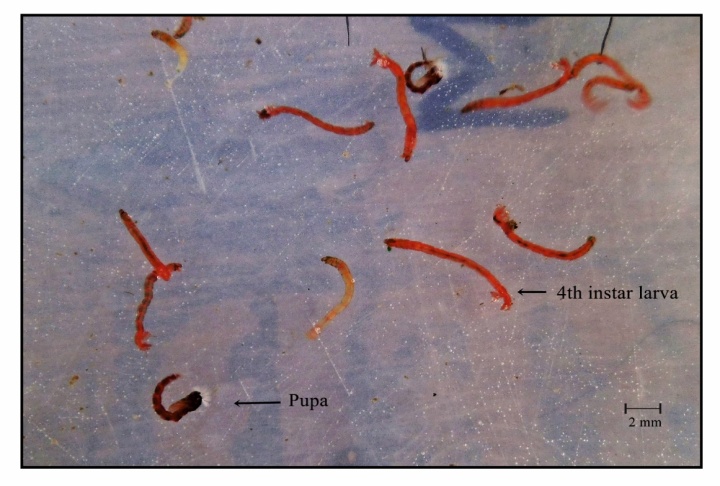


Photo S4: Larvae and pupa


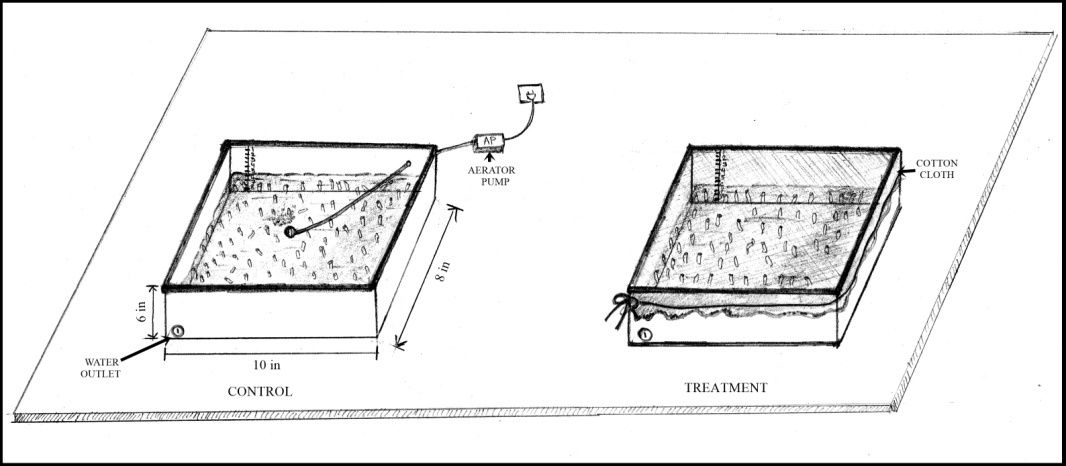


Photo S5: Diagram of Experimental design.
