# Supplementary material for: Tube length of chironomid larvae as an indicator for dissolved oxygen in water bodies
Source: Sci Rep. 2022 Nov 19;12:19971. doi: 10.1038/s41598-022-23953-9 (PMC9675818; doi:10.1038/s41598-022-23953-9)
Supplement: Supplementary file 2 — Supplementary Information 2. [file 41598_2022_23953_MOESM2_ESM.docx]

Table S 1: Table of Stepwise regression (Consider model IV and VI)

| Group | Model |  | β | s.e. | p | Group | Model |  | β | s.e. | p |
| --- | --- | --- | --- | --- | --- | --- | --- | --- | --- | --- | --- |
| 1 | I | Constant | 16.159 | 2.258 | <0.001 | 2 | I | Constant | 14.009 | 1.235 | <0.001 |
|  |  | DO | -1.438 | 0.513 | 0.019 |  |  | DO | -0.929 | 0.177 | <0.001 |
|  |  | R^2^ | 0.440 | | |  |  | R^2^ | 0.734 | | |
|  | II | Constant | 29.683 | 1.28 | <0.001 |  | II | Constant | -0.927 | 4.001 | 0.822 |
|  |  | DO | -2.931 | 0.183 | <0.001 |  |  | DO | -1.708 | 0.235 | <0.001 |
|  |  | Wt_temp | -0.268 | 0.023 | <0.001 |  |  | pH | 2.595 | 0.681 | 0.004 |
|  |  | R^2^ | 0.966 | | |  |  | R^2^ | 0.898 | | |
|  | III | Constant | 27.193 | 1.011 | <0.001 |  | III | Constant | -1.959 | 2.779 | 0.501 |
|  |  | DO | -2.933 | 0.113 | <0.001 |  |  | DO | -1.731 | 0.162 | <0.001 |
|  |  | Wt_temp | -0.205 | 0.021 | <0.001 |  |  | free_CO_2_ | 0.277 | 0.084 | 0.011 |
|  |  | free_ CO_2_ | 0.035 | 0.009 | 0.004 |  |  | pH | 2.054 | 0.498 | 0.003 |
|  |  | R^2^ | 0.989 | | |  |  | R^2^ | 0.957 | | |
|  | IV | Constant | 27.291 | 0.667 | <0.001 |  | IV | Constant | 3.832 | 3.11 | 0.258 |
|  |  | DO | -2.779 | 0.087 | <0.001 |  |  | DO | -2.207 | 0.224 | <0.001 |
|  |  | Wt_temp | -0.215 | 0.014 | <0.001 |  |  | free_CO_2_ | 0.346 | 0.07 | 0.002 |
|  |  | free_CO_2_ | 0.028 | 0.006 | 0.003 |  |  | pH | 0.769 | 0.631 | 0.262 |
|  |  | Nitrite | -1.259 | 0.373 | 0.012 |  |  | TH | 0.046 | 0.018 | 0.038 |
|  |  | R^2^ | 0.996 | | |  |  | R^2^ | 0.978 | | |
|  | | | | | |  | V | Constant | 7.419 | 1.044 | <0.001 |
|  |  |  |  |  |  |  |  | DO | -2.312 | 0.212 | <0.001 |
|  |  |  |  |  |  |  |  | Free CO_2_ | 0.388 | 0.063 | <0.001 |
|  |  |  |  |  |  |  |  | TH | 0.064 | 0.011 | <0.001 |
|  |  |  |  |  |  |  |  | R^2^ | 0.973 | | |
|  |  |  |  |  |  |  | VI | (Constant) | 8.029 | 0.841 | <0.001 |
|  |  |  |  |  |  |  |  | DO | -2.044 | 0.195 | <0.001 |
|  |  |  |  |  |  |  |  | free_CO_2_ | 0.41 | 0.049 | <0.001 |
|  |  |  |  |  |  |  |  | TH | 0.051 | 0.01 | 0.002 |
|  |  |  |  |  |  |  |  | Iron | -1.296 | 0.511 | 0.039 |
|  |  |  |  |  |  |  |  | R^2^ | 0.986 | | |

Table S 2: Pearson’s correlation between different physicochemical parameters

|  |  | Tube length | |
| --- | --- | --- | --- |
|  | Group | I | II |
| DO | r | -0.663* | -0.857** |
|  | p | 0.019 | <0.001 |
| free_CO_2_ | r | 0.136 | -0.174 |
|  | p | 0.673 | 0.590 |
| TA | r | 0.020 | -0.704* |
|  | p | 0.951 | 0.011 |
| TH | r | -0.530 | -0.717** |
|  | p | 0.076 | 0.009 |
| Salinity | r | -0.115 | -0.726** |
|  | p | 0.723 | 0.007 |
| SOC | r | 0.169 | -0.344 |
|  | p | 0.600 | 0.274 |
| pH | r | -0.428 | -0.548 |
|  | p | 0.165 | 0.065 |
| Wt_temp | r | -0.070 | -0.144 |
|  | p | 0.830 | 0.655 |
| Wt_tu | r | 0.416 | -0.487 |
|  | p | 0.179 | 0.108 |
| Wtr | r | -0.059 | 0.606* |
|  | p | 0.855 | 0.037 |
| Ammonia | r | -0.393 | -0.086 |
|  | p | 0.206 | 0.791 |
| Nitrite | r | -0.535 | 0.197 |
|  | p | 0.073 | 0.540 |
| Nitrate | r | -0.144 | -0.441 |
|  | p | 0.655 | 0.152 |
| Phosphate | r | 0.210 | -0.119 |
|  | p | 0.513 | 0.712 |
| Iron | r | -0.637* | -0.494 |
|  | p | 0.026 | 0.102 |

r= Pearson’s correlation value

Table shows Pearson’s correlation analysis between tube length and all other parameters for Group I and II. In group I, tube length is negatively correlated with DO (dissolved oxygen) (r= -0.663, p=0.019) and iron (r= -0.637, p=0.026) and the relationship is statistically significant. In group II, tube length isnegatively correlated with DO (dissolved oxygen) (r=-0.857, p<0.001), ta (r=-0.704, p=0.011), th (r=-0.717, p=0.009) and salinity(r=-0.726, p=0.007) and positively correlated with wtr (r=0.606, p=0.037) and the relationship is statistically significant.

Fig. S 1. Abundance of CS (*Chironomus striatipennis*) at KWC

Fig.S 2. Abundance of CS (*Chironomus striatipennis* ) at KFP

Fig.S3. The PCA biplot showing the ordination of the variables of Physico-chemical parameters and species composition (CS: *Chironomus striatipennis*), (DO: Dissolved oxygen) at KWC.

Fig.S4. The PCA biplot shows the ordination of the variables of Physico-chemical parameters and species composition (CS: *Chironomus striatipennis*) (DO: Dissolved oxygen)at KFP.
